# Editorial: Customized artificial implants: bionic design and multiscale evaluation

**DOI:** 10.3389/fbioe.2024.1425080

**Published:** 2024-05-14

**Authors:** Zhenxian Chen, Yongtao Lyu, Junyan Li, Xijin Hua

**Affiliations:** ^1^ School of Mechanical Engineering, Chang’an University, Xi’an, China; ^2^ School of Mechanics and Aerospace Engineering, Dalian University of Technology, Dalian, China; ^3^ Tribology Research Institute, Southwest Jiaotong University, Chengdu, China; ^4^ Department of Engineering, Faculty of Environment, Science and Economy, University of Exeter, Exeter, United Kingdom

**Keywords:** customized artificial implants, bionic design, multiscale evaluation, biomechanics, computational simulation, experimental testing

Due to individual differences, anatomical variability and complexity of working conditions, traditional “one-size-fits-all” implants cannot fulfill the clinical requirements. With the concept of precision medicine or personalized medicine being proposed and hotly discussed, more surgeons and engineers are designing, manufacturing, evaluating, and applying customized artificial implants. Meanwhile, advanced technologies such as 3D printing and multiscale computer simulations are promoting the development of customized artificial implants. Especially, customized artificial implants are booming in orthopedics and orthodontic surgeries recently. Customized orthopedic implants can offer remarkable precision and fit, improve functionality, reduce pain and inflammation, and accelerate healing. However, challenges to implement precision design and evaluation arise not only from the design need to consider bionic structures, kinematical function, mechanical performances, and biological function but also from the performance and functional evaluation involving multiscale computational simulations and comprehensive experimental testing. There is still a lack of knowledge on customized principles, design methods, evaluation systems, new material applications, and surgical plans. Advancements have been made to delve into the role of bionic design in precision treatment and long-term success, as well as the importance of multiscale evaluation to ensure the safety and efficiency of these life-changing devices.

Irregular bone defects or resection areas are common in orthopedic clinical practice, and anatomical matching design is crucial for artificial implants (Wang et al., 2022). Hu et al. designed and used a 3D-printed custom prosthesis for a patient with irregular humeral defects accompanied by shoulder joint “locking” dislocation and reverse Hill-Sachs injury. Liu et al. reported a 3D-printed integrated acetabular prosthesis and modular acetabular prosthesis for the acetabular reconstruction of total hip arthroplasty in Crowe III hip dysplasia. Wang et al. designed a novel individualized porous titanium alloy zero-profile cage for anterior cervical discectomy and fusion based on the morphological characteristics of the intervertebral space. However, in fact, customized artificial implant not only requires anatomical matching but also need to deeply focus on mechanical matching and kinematical matching. Wang et al. designed an innovative temporomandibular joint (TMJ) fossa prosthesis based on the envelope surface of condyle movement. This customized design of fossa prosthesis not only successfully achieved a wider condylar range of motion but also reduced the muscle activation for jaw opening on the surgical side and resistance on the intact side than traditional commercial implants.

Except for customized orthopedic implants, customized breast implants, dental implants, and stents have been developed and studied. Tong et al. proposed a customized design strategy for the single-tube-braided airway stents to meet various airway structures, investigated the radial stiffness of the stents and deformation upon compression using a theoretical model, and evaluated their mechanical properties and functions using experimental testing. The proposed customized stents adapt well to different airway structures.

The customized implant is costly and time-consuming due to its customized nature (Hafez et al.). Artificial intelligence or machine learning may help to improve the drawbacks of long design cycles, such as quickly obtaining a patient’s bone geometry and mechanical property (Lu et al., 2023), and creating the 3D model of artificial implants (Burge et al., 2023). Triply periodic minimal surface (TPMS) is widely used in the design of bone scaffolds for large bone detects due to its structural advantages. Liu et al. proposed a new inverse design of an anisotropic TPMS bone scaffold based on the mechanical properties of bone structures using machine learning and a regenerative genetic algorithm. Combining machine learning with the traditional optimization method achieves higher design efficiency, and the entire design process is easily controlled.

Multiscale evaluation of customized implants encompasses a range of assessments that consider the implant’s performance and interaction with the body at various levels, from the macroscopic to the microscopic. Musculoskeletal (MSK) multibody dynamics (MBD) model has a remarkable advantage in simulating human macro physical activities and getting joint force and motion, ligament force, muscle force or activation. Wang et al. adopted a mandibular MSK MBD model to evaluate the jaw opening-closing motions, mandibular muscle activation, and contact forces of the customized TMJ fossa prosthesis. The finite element analysis (FEA) method can be used to quantify the stress and strain of artificial implants from a microscopic point of view. Yang et al. analyzed the position, structure, and spread area of the wing fixture of a new customized implant applied in severe atrophic maxillary posterior region restoration using FEA. Combining the advantages of MSK MBD simulation and FEA, the coupling analysis method of both (Zhang et al., 2017; Hua et al., 2022) and the FE MSK MBD model (Li et al., 2019) are the current development trend of multiscale evaluation. Meanwhile, a combination of theoretical models or computer simulation and biomechanical experiments are recommended to comprehensively evaluate the safety, efficacy stability of artificial implants (Li et al.). In addition, clinical case observation and report are equally important for systematically evaluating customized implants (Hu et al.), which should be combined with the aforementioned methods. Clinical trials and patient outcomes provide real-world data that feed back into the design process, ensuring continuous improvement and refinement. The traditional experimental methods and devices do not work on most customized artificial implants. New test standards, technologies, and equipment need to be established and developed for testing the rising customized designs.

Overall, customized artificial implants represent a significant leap forward in medical technology. This Research Topic of articles contributes to promote the development of customized artificial implants in bionic design, multiscale evaluation, and translation. New technology based on machine learning and new materials have been introduced into the precise design of customized artificial implants. Bionic design methods based on joint kinematics and the mechanical properties of bone structures are developed for customized artificial implants. The exploitation and application of novel approaches, testing techniques, and standards in evaluation are still scarce. The challenges remain in the high-efficiency, accurate, and quick design and evaluation of customized artificial implants.
